# Editorial: Immune Tolerance Post Allogeneic Hematopoietic Cell Transplantation

**DOI:** 10.3389/fimmu.2020.00523

**Published:** 2020-03-24

**Authors:** Dominik Schneidawind, Everett Meyer

**Affiliations:** ^1^Department of Medicine II, University Hospital Tübingen, Tübingen, Germany; ^2^Division of Blood and Marrow Transplantation, Stanford University School of Medicine, Stanford, CA, United States

**Keywords:** allogeneic HCT, GvHD, immune tolerance, GVL effects, graft engineering

Within the last decade significant progress has been accomplished in the field of allogeneic hematopoietic cell transplantation (HCT). The articles in this special topics series present progress in improving clinical outcomes using some of the most vibrant current research and translational approaches, including novel reduced-intensity conditioning regimens, donor graft engineering, quantification of the microbiome, and tailored immunosuppressive therapies. Much of the progress described is due to improvements in immune reconstitution that results in durable allograft tolerance. Consequently, there is reduced graft-vs.-host disease (GVHD) and improvement in controlling infectious disease complications. In addition, improved immune reconstitution also appears to facilitate much-needed graft-vs.-leukemia effects, as relapse remains the major challenge of our field. Many of the articles in this special topics series are organized around our increasing understanding of GVHD and a suite of new tools and approaches to prevent and treat this dreaded immune complication.

Thangavelu and Blazar from the University of Minnesota provide an overview of our current understanding of GVHD pathophysiology and thoroughly review novel therapeutic strategies to induce immune tolerance focusing on biologicals, epigenetic modulation, and adoptive cell therapy. In particular, light is shed on the role of the intestinal microbiome for GVHD induction by Köhler and Zeiser: within the last years it became evident in various preclinical and clinical studies that changes of the bacterial composition affects the risk of intestinal GVHD which also constitutes a potential target to prevent deleterious damage of the gut. For patients with steroid-refractory acute and chronic GVHD, extracorporeal photopheresis (ECP) is an established procedure to induce immune tolerance and a significant impact of apoptotic bodies to modulate dendritic-cell function has been established. Ni et al. now suggest that also NK-cell subsets are influenced by ECP in such a way that CD56^high^CD16^−^ NK cells were decreased and cytotoxicity shifted toward a regulatory phenotype while maintaining antileukemic activity.

Efforts to define normal and healthy from abnormal and immune reconstitution that puts recipients at increased risk of GVHD continues to develop with the application of immune monitoring, as illustrated by Soares et al. who performed a prospective comparative analysis that suggests that thymic damage results in dysfunctional thymic output with increased CD8^+^ terminally differentiated effector memory T cells and decreased T-cell receptor diversity. This study emphasizes the thymus as critical organ for central immune tolerance during immune reconstitution and sustained immune tolerance after allogeneic HCT. Simonetta et al. performed a comprehensive analysis of PD-1 expression on T cells following allogeneic HCT noticing an increase early after transplantation without impaired production of cytotoxic effector molecules. This study provides insight into dynamic T-cell regulation also suggesting that timing should be considered when check point inhibitors are applied.

Efforts to engineer donor grafts have shown evidence in pre-clinical and clinical studies of improved immune reconstitution. Bertaina and Roncarolo from Stanford University review such approaches focusing on T- and B-cell depletion strategies as well as regulatory T cells. In particular, three papers included in this Research Topic explore double-negative T cells, myeloid-derived suppressor cells (MDSCs) and invariant natural killer T (iNKT) cells for GVHD prevention. Haug et al. found that TCRαβ^+^CD4^−^CD8^−^ T cells inhibit mammalian target of rapamycin (mTOR) signaling and prevent metabolic adaption of conventional T helper cells resulting in decreased homing receptor expression and production of proinflammatory cytokines. The expansion of MDSCs from hematopoietic stem cells has been studied by Park et al. showing that these cells retain a suppressive phenotype and ameliorate GVHD in a xenogeneic GVHD model also resulting in improved survival. Jahnke et al. demonstrate that human iNKT cells that have been shown to promote immune tolerance after allogeneic HCT can also be expanded from cryopreserved donor lymphocytes efficiently lysing patient AML blasts.

Finally, two review articles provide detailed insights into innovative approaches of immune tolerance induction. Stahl et al. summarize preclinical and clinical data about the CD4 antibody MAX.16H5 that has been investigated in auto- and alloimmunity. Wajant and Beilhack from Würzburg highlight the impact of tumor necrosis factor signaling on the regulation of FoxP3 regulatory T cells being known as central players for sustained immune tolerance after allogeneic HCT.

This Research Topic bundles cutting-edge, innovative, and translational original research articles as well as excellent reviews from renowned scientists highlighting recent progress in the field of transplant immunology and immune tolerance.

## Author Contributions

DS and EM wrote the editorial.

### Conflict of Interest

The authors declare that the research was conducted in the absence of any commercial or financial relationships that could be construed as a potential conflict of interest.

